# Difficulties Faced by Undergraduates While Conducting Endodontic Therapy

**DOI:** 10.7759/cureus.52217

**Published:** 2024-01-13

**Authors:** Nawaf Almutairi, Abdullah Alharbi

**Affiliations:** 1 Conservative Dental Sciences and Endodontics, College of Dentistry, Qassim University, Qassim, SAU

**Keywords:** survey, student perception, endodontic training, endodontic curriculum, endodontic difficulties

## Abstract

Background: Dentistry is a complex field that utilizes both theoretical and practical knowledge along with a distinct coordination of the hand, brain, and eye of an individual, forming a major part of endodontic therapy. Assessing the understanding and practical knowledge of undergraduates from time to time is essential in evaluating the success of the undergraduate education system. Therefore, the purpose of this study was to evaluate the challenges that are associated with endodontic therapy and are faced by undergraduate students at College of Dentistry, Qassim University.

Methodology: Ninety-seven undergraduates who had completed their preclinical training and had done endodontic treatments were included in this questionnaire-based study. The Chi square test and Fisher's exact test have been used to determine whether or not there is a significant difference on a categorical scale between two or more groups of study parameters.

Results: According to our results, de-roofing of the pulp chamber and recognizing the canal orifices are the two areas that need more consideration. The gender-wise difference in the challenges faced by the students while performing the majority of steps during endodontic treatment was found to be insignificant. However, a significant difference was noted in relation to the complete deroofing of the pulp chamber (p-value=0.04) and locating the canal orifices (p-value=0.04). Moreover, fifth year students rarely faced difficulties in performing anesthesia and rubber dam placement, whereas fourth year students faced difficulties in both procedures.

Conclusion: The study concludes that de-roofing the pulp chamber and identifying the orifices of canals are two areas where more attention should be given while training undergraduate dental students.

## Introduction

The successful execution of all root canal treatment methods is dependent upon their correct application. Theoretical and practical clinical training in endodontics forms a crucial component of the undergraduate dental program, and students should gain the skills necessary to execute simple root canal procedures during their undergraduate training [1]. The assessment of undergraduate students is an essential part of the process of developing new instructional strategies [2]. Learning and performance can be influenced by a wide variety of factors, including clinical experience, relationships between teachers, students, and patients, clinical application of theory, extracurricular activities, student effort, and self-confidence [3]. The opinions of students can help with curriculum assessment, leading to a higher quality of instruction and better treatment for patients [4].

Endodontic discipline, in various stages of endodontic treatment and diagnosis, makes an effort to develop knowledge of tactile perception of both the external surface and interior tooth anatomy. As root canal anatomy is varied and complex anatomically, the majority of students and practitioners feel unprepared to perform several procedures, especially endodontic therapy of the molars. Dental students face a number of hurdles throughout their training, including a lack of self-confidence, anatomical variances, and personal responsibility for the provision of adequate health care to patients [5].

Self-efficacy is a term that relates to the sentiments of competence and confidence that one possesses, described as the self-assurance that one is able to successfully do certain activities. Self-efficacy requires one to possess a certain level of competence [6]. There has not been enough research done in Saudi dental schools on the level of self-efficacy that general dentistry practitioners have in connection to endodontics. In addition, there is not a lot of information available regarding the self-efficacy of undergraduate dentistry students [7]. Hence, this study aimed to recognize and evaluate the difficulties encountered during endodontic therapy by undergraduate dental students during various stages of endodontic therapy (achieving profound anesthesia, using a rubber dam, taking a radiograph, accessing the cavity, de-roofing, identifying the number of root canal orifices, and using the lateral condensation technique) at the College of Dentistry, Qassim University.

## Materials and methods

The present cross-sectional study was carried out among dental clusters at the College of Dentistry at Qassim University. Ethical clearance was obtained from the institutional ethical committee (#EA.M-2020-3005) of the College of Dentistry, Qassim University, and informed consent was also obtained from each participant.

Undergraduate dental students studying in the fourth and fifth years who had completed the preclinical course in endodontics were included in the study. While students who did not practice endodontic treatment OR belonged to other universities were excluded. 

The sample size was calculated by utilizing the Qualtrics sample size calculator. A sample of 86 respondents was calculated as an adequate sample size with a 5% margin of error, a 95% confidence interval, and a total target student population of 110 [8]. A study-specific questionnaire was used in the pilot study that was based on a previous study [3,4] by Tavares et al. (2018) and Mirza MB et al. (2015) with minor modifications regarding details of procedures performed during endodontic treatment in order to assess the difficulties, performance, and challenges that dental students face when it comes to endodontic treatment. This questionnaire was then tested with a group of 30 participants. Both the responses and comments made by this group were read by two professors who were experts in the design of questionnaires in order to evaluate and improve the instrument. The format underwent some minor adjustments, and coding was completed.

Dentistry is a five-year program in Saudi Arabia. Students study endodontics as part of the preclinical course in the third academic year, during which they also receive training in the lab on both extracted and artificial teeth. While students in their fourth academic year perform straightforward endodontic treatments (on single-rooted or two-rooted teeth), students in their fifth academic year tackle more difficult endodontic procedures, such as those involving multirooted teeth. This study included participation from a total of 97 dental undergraduate students who were currently enrolled in endodontics classes. An electronic questionnaire was used via "Google Forms" and was sent electronically to the students. The responses to the electronic questionnaire were compiled in an Excel sheet, and the data were decoded using an independent investigator.

Statistical analysis

IBM Corp. Released 2017. IBM SPSS Statistics for Windows, Version 25.0. Armonk, NY: IBM Corp. was used to perform the analysis on the data. The Chi square test and Fisher's exact test were used to determine whether or not there is a significant difference on a categorical scale between two or more groups of study parameters. When the value of p was less than 0.05 at the 95% confidence interval, statistical significance was considered (CI).

## Results

A total of 97 students in their fourth and fifth years participated in this cross-sectional study. The participants were split evenly between the sexes. A total of 97 responses were gathered, with 61 responses coming from male students and 36 responses coming from female students. A total of 59 students from the fourth year and 38 students from the fifth year responded to the survey.

According to the findings of our research, there were no statistically significant differences between male and female students in the following steps: performing anesthesia, using a rubber dam, taking X-rays or performing the same lingual opposite buccal (SLOB) technique, access cavity preparation, and using the lateral condensation technique (Table 1).

**Table 1:** The distribution of responses between male and female participants in the study with the statistical correlation.

			1- I face difficulty in preforming anesthesia during endodontic treatment				Total	X2	P-value
			Frequent	Never	Rare	Sometime			
		Count	7	8	25	21	61	4.615	.202
Gender:	Male	% of Total	11.5%	13.1%	41.0%	34,4%	100.0%		
		Count	3	10	9	14	36		
Gender:	Female	% of Total	8.3%	27.8%	25.0%	38.9%	100.0%		
			2- I have difficulty in the use of rubber dam				Total	X2	P-value
			Frequent	Never	Rare	Sometime			
		Count	7	8	18	28	61	1.329	.722
Gender:	Male	% of Total	11.5%	13.1%	29.5%	45.9%	100.0%		
		Count	4	5	7	20	36		
Gender:	Female	% of Total	11.1%	13.9%	19.4%	55.6%	100.0%		
			3- I have a difficulty in taking an X-ray during endodontic treatment				Total	X2	P-value
			Frequent	Never	Rare	Sometime			
		Count	9	7	13	32	61	6.292	.098
Gender:	Male	% of Total	14.8%	11.5%	21.3%	52.5%	100.0%		
		Count	9	8	9	10	36		
Gender:	Female	% of Total	25.0%	22.2%	25.0%	27.8%	100.0%		
			4- I have difficulty in using SLOB radiographic technique used in endodontic treatment				Total	X2	P-value
			Frequent	Never	Rare	Sometime			
		Count	14	5	15	27	61	2.445	.485
Gender:	Male	% of Total	23.0%	8.2%	24.6%	44.3%	100.0%		
		Count	8	2	14	12	36		
Gender:	Female	% of Total	22.2%	5.6%	38.9%	33.3%	100.0%		
			5- I am facing difficulty during endodontic access cavity preparation				Total	X2	P-value
			Frequent	Never	Rare	Sometime			
		Count	0	7	32	22	61	6.189	.103
Gender:	Male	% of Total	0%	11.5%	52.5%	36.1%	100.0%		
		Count	2	2	14	18	36		
Gender:	Female	% of Total	5.6%	5.6%	38.9%	50.0%	100.0%		

			6- I have difficulty to make sure that the roof the pulp chamber is completely de-roofed				Total	X2	P-value
			Frequent	Never	Rare	Sometime			
		Count	4	6	33	18	61		
Gender:	Male	% of Total	6.6%	9.8%	54.1%	29.5%	100.0%	7.886	.048
		Count	4	5	9	18	36		
Gender:	Female	% of Total	11.1%	13.9%	25.0%	50.0%	100.0%		
			7- I have difficulty in identifying the number of the orifices				Total	X2	P-value
			Frequent	Never	Rare	Sometime			
		Count	5	1	18	37	61		
Gender:	Male	% of Total	8.2%	1.6%	29.5%	60.7%	100.0%	7.981	.046
		Count	9	2	5	20	36		
Gender:	Female	% of Total	25.0%	5.6%	13.9%	55.6%	100.0%		
			8- I have difficulty in using lateral condensation technique				Total	X2	P-value
			Frequent	Never	Rare	Sometime			
		Count	4	7	22	28	61		
Gender:	Male	% of Total	6.6%	11.5%	36.1%	45.9%	100.0%	2.433	.488
		Count	4	7	13	12	36		
Gender:	Female	% of Total	11.1%	19.4%	36.1%	33.3%	100.0%		

With respect to questions six and seven, there were significant gender differences in terms of the level of difficulty experienced by male and female students. 29.5% of male students and 50% of female students sometimes had difficulties ensuring the deroofing of the pulp chamber. In addition, 8.2% of male students and 25% of female students frequently experienced difficulties in identifying the numbers of orifices.

According to the findings, there are no significant differences in the levels of difficulty experienced by students in their fourth and fifth years when it comes to the following steps of endodontic treatment: using the SLOB radiographic technique, access cavity preparation, complete de-roofing of the pulp chamber, and using the lateral condensation technique (Table 2).

**Table 2:** The distribution of responses between fourth and fifth year students included in the study and the statistical correlation.

			1- I face difficulty in preforming anesthesia during endodontic treatment				Total	X2		P-value
			Frequent	Never	Rare	Sometime		8.383a	0.039	
		Count	0	8	17	13	38			
Academic Year:	5^th^	% of Total	0%	21.1%	44.7%	34.2%	100%			
		Count	10	10	17	22	59			
Academic Year:	4^th^	% of Total	16.9%	16.9%	28.8%	37.3%	100%			
			2- I have difficulty in the use of rubber dam				Total	X2		P-value
			Frequent	Never	Rare	Sometime		8.687	0.034	
		Count	1	3	14	20	38			
Academic Year:	5^th^	% of Total	2.6%	7.9%	36.8%	52.6%	100%			
		Count	10	10	11	28	59			
Academic Year:	4^th^	% of Total	16.9%	16.9%	18.6%	47.5%	100%			
			3- I have a difficulty in taking an x-ray during endodontic treatment				Total	X2		P-value
			Frequent	Never	Rare	Sometime				
		Count	6	7	15	10	38	12.541		.006
Academic Year:	5^th^	% of Total	15.8%	18.4%	39.5%	26.3%	100%			
		Count	12	8	7	32	59			
Academic Year:	4^th^	% of Total	20.3%	13.6%	11.9%	54.2%	100%			
			4- I have difficulty in using SLOB radiographic technique used in endodontic treatment				Total	X2		P-value
			Frequent	Never	Rare	Sometime				
		Count	7	4	15	12	38	4.521		.210
Academic Year:	5^th^	% of Total	18.4%	10.5%	39.5%	31.6%	100%			
		Count	15	3	14	27	59			
Academic Year:	4^th^	% of Total	25.4%	5.1%	23.7%	45.8%	100%			
			5- I am facing difficulty during endodontic access cavity preparation				Total	X2		P-value
			Frequent	Never	Rare	Sometime				
		Count	1	6	21	10	38	7.136		.068
Academic Year:	5^th^	% of Total	2.6%	15.8%	55.3%	26.3%	100%			
		Count	1	3	25	30	59			
Academic Year:	4^th^	% of Total	1.7%	5.1%	42.4%	50.8%	100%			

			6- I have difficulty to make sure that the roof the pulp chamber is completely de-roofed				Total	X2	P-value
			Frequent	Never	Rare	Sometime			
		Count	1	4	16	17	38		
Academic Year:	5^th^	% of Total	2.6%	10.5%	42.1%	44.7%	100%	3.424	.331
		Count	7	7	26	19	59		
Academic Year:	4^th^	% of Total	11.9%	11.9%	44.1%	32.2%	100%		
			7- I have difficulty in the number of identifying the orifices				Total	X2	P-value
			Frequent	Never	Rare	Sometime			
		Count	1	3	4	30	38		
Academic Year:	5^th^	% of Total	2.6%	7.9%	10.5%	78.9%	100%	19.598	.000
		Count	13	0	19	27	59		
Academic Year:	4^th^	% of Total	22.0%	0%	32.2%	45.8%	100%		
			8- I have difficulty in using lateral condensation technique				Total	X2	P-value
			Frequent	Never	Rare	Sometime			
		Count	5	5	13	15	38		
Academic Year:	5^th^	% of Total	13.2%	13.2%	34.2%	39.5%	100%	2.005	.571
		Count	3	9	22	25	59		
Academic Year:	4^th^	% of Total	5.1%	15.3%	37.3%	42.4%	100%		

In contrast, only 44.7% of students in the fifth year encounter difficulties in performing anesthesia, while 37.3% of students in the fourth year are occasionally confronted with challenges in the administration of local anesthesia. 36.8% of students in the fifth year very rarely had difficulty using a rubber dam, whereas 47.5% of students in the fourth year have encountered this difficulty sometimes. In addition, 39.5% of students in their fifth year experience difficulties taking radiographs, while 54.2% of students in their fourth year experience difficulties occasionally. A total of 78.9% of students in their fifth year occasionally struggled to identify the number of orifices, while only 32.2% of students in their fourth year rarely experienced such difficulties. 

## Discussion

The study of dentistry requires a significant amount of hand, eye, and brain coordination, including a specific magnitude of manual dexterity that is not seen in other fields. Many dental students, especially those performing molar endodontic treatment, view endodontics as a difficult and stressful field of study due to the structural variation of root canals, the need to provide satisfactory quality care, and low self-esteem [5]. The behavior that is anticipated of newly trained individual practitioners is defined as "clinical competency." This conduct takes into account an individual's knowledge, abilities, and morals in order to formulate a holistic reaction to the diverse array of situations typically encountered in ordinary professional activity [9,10].

Taking into account the opinions of current and former students is the most effective way to evaluate the standard of education offered at any given establishment [11]. Also, one of the most important factors in healthy student growth is a productive relationship between students and teachers [12,13]. Unfortunately, the perspectives of the students are often disregarded, particularly when planning for curriculum modifications [4]. Hence, this study was undertaken to assess the difficulties arising among the students with respect to endodontic training.

In the present study, comparisons were made between the genders with regard to several steps in endodontic therapy based on a questionnaire filled out by fourth and fifth year students. There were no significant differences noted between males and females in various steps, including performing anesthesia, rubber dam application, taking a radiograph or using the SLOB technique, access cavity preparation, and using the lateral condensation technique. But, significant differences were seen within sexes with respect to making sure that the roof of the pulp chamber was completely deroofed (p<0.05). About 25% of females faced difficulty rarely, as compared to 54.1% of males. Additionally, 13.9% of females faced difficulty identifying the numbers of orifices, as compared to 29.5% of males (p<0.05). These differences could be attributed to the fact that there was lower participation from the female students as compared to the male students.

Root canal therapy can only be considered successful if both the root canal space and the coronal part of the pulp cavity are thoroughly cleaned [14]. This can only be accomplished by creating an ideal access cavity and subsequent de-roofing. In a study done by Kaplan et al. [15], they discovered that students who struggled to open access cavities also struggled to remove the pulp chamber roof, making it hard for them to identify root canals. This was due to the fact that opening access cavities requires removing the pulp chamber roof.

Mirza et al. [4] found that the majority of students struggled with patient management, local anesthetic administration, and the use of rubber dams. Whereas in the present study, most of the students faced difficulty sometimes in rubber dam application, administration of local anesthesia, use of SLOB, number of orifices, and use of lateral condensation technique, but the results were not statistically significant.

According to Kaplan et al. [15], more than half of the students had difficulty while attempting to do periapical radiography. When taking periapical radiographs, one of the most common mistakes that students make is having incorrect angulation in relation to anatomical locations, as mentioned by Peker and Alkurt in 2009 [16]. This is one of the most common problems encountered and can be corrected by demonstrating the theory with the use of practical models and apex locators.

According to the results of the present study, the fourth- and fifth-year students displayed no significant differences in the difficulties in the use of the SLOB radiographic technique, access cavity preparation, complete de-roofing of the pulp chamber, or using the lateral condensation technique. But, 44.7% of fifth-year students rarely faced difficulties in performing anesthesia, while 37.3% of fourth-year students sometimes faced difficulties. Also, 36.8% of fifth-year students rarely had difficulties with the use of rubber dams, whereas 47.5% of fourth-year students sometimes had. Additionally, 39.5% of fifth graders rarely had difficulties taking radiographs, while 54.2% of fourth graders sometimes faced difficulties. 78.9% of fifth-year students sometimes had difficulty identifying the number of orifices, while 32.2% of fourth-year students rarely faced difficulties. Our findings are corroboration with the qualitative research done by Seijo et al. [17], who found that students had problems with the following aspects of dental care: specific techniques, including radiograph exposure, rubber dam placement, cavity access, root canal exploration, instrumentation, and filling, and the treatment of curved and narrow canals. Kaplan et al. [15] reported that working length determination requires taking periapical radiographs with an initial apical file, so students who struggled with this skill also struggled with determining working length. According to Tavares et al. [3], the education approach should place a stronger emphasis on radiographic techniques and the selection of intracanal medicine, which was based on a survey-based study done at a Brazilian university. In addition to this, there is a pressing requirement to heighten students' awareness of prior theoretical knowledge as well as the duties that fall on their shoulders throughout therapy [3,18,19].

It is possible to address these issues by incorporating the removal of teeth affected by caries into the preclinical training of students. In addition, diagnostic radiographs are required to be used in endodontic clinics to examine not only the root canals and the periapical area, but also the crown of the tooth in order to determine the extent to which carious lesions have spread and the limits of the pulp chamber. This is done in order to determine the spread of carious lesions [20,21]. This is necessary in order to properly diagnose and treat patients. It is usual practice to center a dental student's learning experience on the teaching crew, the members of which have the responsibility of passing on both theory and practical knowledge and abilities depending on their clinical training. Dental students need to understand why they are learning specific topics in order to be able to make decisions that are applicable to real-world scenarios so that they can effectively acquire the knowledge and skills they are being taught [22].

The limitations of this study were that the findings were based on a specific time frame and a particular set of participants, limiting the generalizability of the results. Additionally, external factors, such as variations in curriculum and teaching methods among dental schools, may influence the applicability of the findings on a broader scale. Furthermore, the study primarily relies on self-reported data, which may be subject to participant bias. Future research should consider a more diverse sample and incorporate objective measures to enhance the robustness of the findings.

## Conclusions

Recognizing the significant student challenges during endodontic treatment procedures enables us to design preclinical and clinical pedagogical approaches. According to our findings, de-roofing the pulp chamber and identifying the orifices of canals are two areas that need to be focused on during training methods. Also, it is necessary to improve student awareness of didactic knowledge and their responsibilities during treatment.
